# Simulation debriefing: a perspective from emergency medical care students at three South African Higher Education Institutions

**DOI:** 10.11604/pamj.2021.38.97.23009

**Published:** 2021-01-28

**Authors:** Andrew William Makkink, Devin John Dreyer

**Affiliations:** 1 Department of Emergency Medical Care, University of Johannesburg, Doornfontein, Johannesburg, South Africa

**Keywords:** Simulation, debriefing, feedback, student perspective

## Abstract

**Introduction:**

simulation-based learning (SBL) is an educational technique that is used to create lifelike experiences within a controlled setting. Feedback and debriefing have been described as most important components in healthcare simulation. Providing feedback or debriefing loses its efficacy if it is not performed correctly. The results of poor feedback or debriefing practice may negatively affect future student performance. It is important to identify both positive and negative current practice so as to better understand the potential effects on student learning. There is a paucity of evidence relating to debriefing within the resource-constrained environment.

**Methods:**

a cross-sectional design collected data using a purpose-designed, paper-based questionnaire that was validated using a pilot study. We collected data from three South African higher education institutions (HEI) offering emergency medical care qualifications. Questionnaires were distributed on-site at each HEI by an academic staff member and were returned to the researchers via courier. Participants were recruited from the second, third and fourth academic years of study. Responses were captured manually and imported into Microsoft Excel for analysis.

**Results:**

we collected 153 completed questionnaires from three South African Higher Education Institutions (HEIs). Student perceptions of debriefing practices were generally positive. There were general feelings of psychological safety and an understanding between students and facilitator related to why debriefing took place. Linking debrief and learning outcomes was perceived as making debriefing meaningful to students. Question-asking techniques by facilitators were mixed, but were generally asked in a manner that encouraged self-reflection. Peer-led debriefing was perceived as good practice and a single facilitator was preferred to multiple facilitators.

**Conclusion:**

several strategies related to effective feedback and debriefing were identified by the student participants as already being employed by facilitators. The potentially negative effect of multiple facilitators was highlighted by participants who indicated that they preferred a single debriefer for the entire academic year. Peer-led debriefing was perceived as a positive practice and has a number of advantages and disadvantages that should be considered and mitigated by the facilitator.

## Introduction

Simulation-based learning (SBL) is an educational technique that is used to create lifelike experiences within a controlled setting. During simulation, learners are able to experience a lifelike and realistic clinical environment in which they can repeatedly practice both technical and non-technical skills with the aim of improving their competence and confidence. Using simulation to teach may not necessarily mean that there is effective learning [1]. Kolb´s theory on experiential learning surmises that effective learning is the result of a learner having an experience and then reflecting on that experience [2]. This reflection gives rise to behavior modification or new ideas and ways of doing things and ultimately to testing the new knowledge. Without a process of reflection, it is unlikely that a learner would identify areas requiring improvement and may, as a result, fail to achieve the relevant learning outcomes [2]. In SBL where certain outcomes may not be obvious to the learner, it is necessary for a facilitator to guide the reflection process by providing feedback or to debrief the student´s performance. Feedback and debriefing have been described as most important components in healthcare simulation [3]. Feedback and debriefing are words that are often, and incorrectly, used interchangeably. Feedback has been defined as “information about performance provided to simulation participants with the intent to modify thinking and/or behavior to facilitate learning and improve future performance” and is therefore considered a one-way transfer of information to the learner [1]. Debriefing on the other hand, has been defined as “an interactive, bidirectional, and reflective discussion or conversation” and is more of a facilitated reflective conversation [1].

In the same way that using simulation does not necessarily mean that there is effective learning, so too, simply providing feedback or debriefing loses its efficacy it is not performed correctly. The results of poor feedback or debriefing practice may negatively affect future student performance. It is important to identify both positive and negative current practice so as to better understand the potential effects on student learning. Three of the primary elements we identified for effective debrief in our environment included that the debrief should be clear and easy to understand, relevant and meaningful and must be constructive and encouraging [4-6]. The format of the debrief is often guided by the aims of the simulation and may focus primarily on technical clinical aspects, on non-technical skills, or a combination of both [7]. Debriefs can be provided by one or more persons and there have been three types of facilitators identified; instructors, peers and the learner themselves [8]. Despite there being a number of guidelines related to debriefing, there is a paucity of literature that explores the perspectives of healthcare students within the resource-constrained environment related to debriefing. Data related to student perspectives on feedback and debriefing may help guide educators in optimizing their own practice. The employment of positive practice and mitigation of negative practices have the potential to optimize student learning. The aim of this study was to explore student perceptions of current feedback and debriefing practice at three South African higher education institutions. This data was used to determine positive and negative practices that had the potential to facilitate or impair teaching and learning practices in the SBL domain.

## Methods

**Study design:** this study used a cross-sectional design to collect data using a purpose-designed, paper-based questionnaire.

**Instrument:** the questionnaire was purpose-designed de novo by the researchers using existing literature as a guide. A pilot study was used to address reliability and validity relating to the questionnaire [9]. Ten first year students at one of the HEIs were used to validate the questionnaire using a pilot study. Pilot study participants were asked to complete the questionnaire and to provide any feedback to the researcher. Prior to completing the pilot study questionnaire, participants were provided a verbal brief and were required to complete a consent form. After the pilot study participants had completed their questionnaires, the researcher held a short discussion session where any issues related to grammar or formatting were discussed. There were no issues identified by the pilot study participants related to understanding or completing the questionnaire. Pilot study responses were not included in the dataset.

**Data collection and setting:** the relevant heads of department were contacted at each of the four respective South African HEIs offering emergency medical care qualifications for permission to approach their students. Three of the four HEIs provided permission and the relevant gatekeeper and ethical approval was granted by each institution prior to data collection commencing. Questionnaires were printed and couriered to each HEI using an approved courier service. Questionnaires were packed by academic year for distribution at each HEI. We included a checklist in each package that explained the procedures that we required the data collector to follow. The package also contained a return envelope for the completed questionnaires to be placed in that was then collected by the courier for return delivery. Each data collector was required to complete a confidentiality document in which they committed to keeping information confidential and to not viewing the responses nor to permitting anyone else to view the responses. Students in the first year of academic study were excluded from the sample. At the time of data collection, first year students would have had very little experience related to SBL. We postulated that their limited experience of SBL would have meant that they would be unable to provide sufficient feedback on the topics under investigation. Questionnaires were distributed at each HEI studied by the relevant data collector. Although we had access to the total number of registered students per HEI, data collectors did not record the number of potential participants present in each data collection session so we were unable to calculate a response rate. Each participant present received an information document related to the study and its aims. Participants were informed of their ability to withdraw at any time without consequence up to the point where they submitted their questionnaire after which it would be impossible to trace the questionnaire due to the anonymous nature of the data. Participants were given a questionnaire to which a consent form had been attached with a paper clip. Participants completed the consent document and questionnaire separately in a classroom setting and then handed them in. Completed documentation was collected, placed into the courier package and returned to the principal investigator.

**Data analysis:** data were manually captured from the paper-based questionnaires into a Microsoft Excel® (Microsoft Office, Microsoft Corporation, Redmond, WA) spreadsheet by DD. Ten percent of the questionnaires captured were checked by AM for accuracy of capture. There were no errors noted. Data were analysed by frequency per question and grouped according to the categories identified and reported on as such.

**Ethical approval:** the study was approved by the University of Johannesburg´s Faculty of Health Sciences Research Ethics Committee (Ref: REC-01-84-2017) prior to any data collection being undertaken. Ethical permission was also granted by the relevant gatekeepers or ethical committees at the three HEIs whose students were invited to participate in the study. We acknowledge that students are a vulnerable population, but the nature of this study meant that these were the only persons who were able to provide the relevant information.

## Results

A total of 153 completed questionnaires were collected from the three HEIs surveyed. Given the nature of the data collection, we were unable to determine response rates. Table 1 depicts the demographics of the respondents. Ninety-one (59.5%) of participants were male and 62 (40.5%) were female. Fifty-six (36.6%) participants were from the second year of academic study, 56 (36.6%) participants were from the third year of academic study and 41 (26.8%) of participants were from the fourth year of academic study.

**Table 1:** demographics of participants from three South African Higher Education Institutions

Academic year of study	Participants N=153	Gender		Mean age in years (IQR)*
		Male N = 91	Female N = 62	
	n (%)	n (%)	n (%)	
Second year	56 (36.6)	35 (62.5)	21 (37.5)	23.6 (5.25)
Third year	56 (36.6)	33 (58.9)	23 (41.1)	24.2 (4.25)
Fourth year	41 (26.8)	23 (56.1)	18 (43.9)	25.5 (5.00)
Total	153 (100)	91 (59.5)	62 (40.5)	24.3 (5.00)

* IQR= Interquartile range

**Essential elements and conversational techniques:** responses were grouped into three primary categories. Two of these categories were described by Sawyer *et al*. namely: essential elements and conversational techniques [1]. Table 2 depicts the responses linked to the two categories described by Sawyer *et al*. Participants indicated that there was a general feeling of psychological safety and that there was generally a common understanding between students and the facilitator related to why debriefing took place. There was general agreement that the facilitator explained the purpose of the simulation and the relevant learning objectives. Participants indicated that debriefing generally addressed learning objectives in a manner that allowed them to compare their performance with the required objectives or outcomes. Facilitators generally focused on the objectives of the simulation as opposed to focusing on technical aspects of the simulation. There was mixed feedback related to how facilitators asked question with the greatest number of participants indicating that facilitators were not inclined to ask simple questions that required “Yes” or “No” answers. Generally, participants indicated that questions were asked in a manner that encouraged self-reflection and the provision of meaningful answers to questions related to why they did specific things. There were mixed perceptions related to how directive feedback the feedback was with slightly more than half of the participants indicating that facilitators tended to focus on technical points and small things related to how skills had been performed.

**Table 2:** essential elements of debriefing as described by Sawyer *et al*.

Question		Strongly Disagree n (%)	Disagree n (%)	Neutral/unsure n (%)	Agree n (%)	Strongly Agree n (%)
**Before debriefing**						
Psychological safety	Generally, I feel that the debriefing environment is one of trust.	0 (0.0)	12 (7.8)	33 (21.6)	88 (57.5)	20 (13.1)
Debriefing stance or basic assumption	Generally, there is a common understanding between students and the facilitator about why debriefing takes place.	3 (2.0)	9 (5.9)	23 (15.0)	96 (62.7)	22 (14.4)
**During debriefing**						
Establishing a shared mental model	Generally, the facilitator explains the purpose of the simulation and how this relates to the learning objectives and the process of debriefing.	2 (1.3)	5 (3.3)	22 (14.4)	90 (58.8)	34 (22.2)
Addressing learning objectives	Generally, the manner in which debriefing takes place allows me to compare my actual performance with the predetermined objectives or outcomes that were required for the simulation.	3 (2.0)	20 (13.1)	18 (11.8)	86 (56.2)	26 (17.0)
	Generally, the facilitator focusses on the objectives of the simulation and not on technical aspects of performing skills.	1 (0.7)	25 (16.3)	49 (32.0)	67 (43.8)	11 (7.2)
Asking open-ended questions	Facilitators are inclined to ask simple questions that require a “Yes” or “No” response during debriefing a simulation.	12 (7.8)	49 (32.0)	53 (34.6)	32 (20.9)	7 (4.6)
**Conversational techniques/educational strategies**						
Learner self-assessment	Generally, the facilitator asks me questions in a manner that encourages me to reflect on what I did during the simulation and to provide meaningful answers about why and how I did what I did.	1 (0.7)	12 (7.8)	16 (10.5)	81 (52.9)	43 (28.1)
Directive feedback	Generally, the facilitator focusses on technical points and small things related to how I performed certain skills.	1 (0.7)	27 (17.6)	45 (29.4)	70 (45.8)	10 (6.5)

**Primary elements related to effective feedback:** the third category related to three primary elements related to effective feedback. The responses linked to the primary elements related to effective debrief are depicted in Table 3. Generally, participants indicated that the feedback of debrief that they were given was easy to understand and that it was relevant and meaningful in that it was sufficient to identify areas requiring improvement. Participants indicated that they were generally positive about debriefing and that they were not embarrassed after having completed a simulation. There were mixed responses related to whether the facilitator focused more on what the learner did right or wrong.

**Table 3:** three primary elements related to effective feedback in our environment

Essential debriefing element	Question	Strongly disagree n (%)	Disagree n (%)	Neutral/unsure n (%)	Agree n (%)	Strongly Agree n (%)
Clear and easy to understand	The feedback/debrief that I get after a simulation assessment is easy to understand.	1 (0.7)	19 (12.4)	25 (16.3)	89 (58.2)	19 (12.4)
Relevant and meaningful	The feedback/debrief that I get after a simulation assessment is sufficient for me to identify areas requiring improvement.	5 (3.3)	24 (15.7)	19 (12.4)	77 (50.3)	28 (18.3)
Constructive and encouraging	I am generally positive about debriefing and feel that it is an important part of simulation-based learning.	2 (1.3)	7 (4.6)	7 (4.6)	75 (49.0)	62 (40.5)
	The way debriefing is conducted makes me feel embarrassed after I have completed a simulation.	27 (17.6)	68 (44.4)	36 (23.5)	19 (12.4)	3 (2.0)
	Generally, the facilitator tends to focus more on what I did wrong rather than focusing on what I did right.	7 (4.6)	37 (24.2)	56 (36.6)	41 (26.8)	12 (7.8)

**Additional questions:** eighty-two (53.6%) participants agreed and 37 (24.2%) strongly agreed that it was good practice to involve fellow students in providing feedback on simulations. Twenty-two (14.4%) disagreed that peer-feedback was good practice in SBL. Eighty-two (53.6%) participants agreed and 32 (20.9%) strongly agreed that actions that they performed that were considered good practice, but not precisely according to the outcomes, were linked to positive feedback that encouraged them to perform the same way again. One-hundred ten (72%) participants indicated that they would prefer the same facilitator providing the debrief for the whole academic year. Forty-three (28%) participants indicated that they would prefer different facilitators teaching and providing feedback during the academic year. Given the option of having different facilitators, 112 (73%) participants indicated that they would prefer each facilitator being allocated a set period of time. In other words, only one facilitator for a month or two after which someone else would take over. The remaining 41 (27%) participants indicated that they would have preferred facilitators teaching interchangeably at the same time.

## Discussion

The aim of this study was to explore student perceptions of current feedback and debriefing practice at three South African higher education institutions. Feedback and debriefing are essential components of effective learning within the SBL domain. This study found that debriefing within three HEIs that offer emergency medical care qualifications appear to adhere to many of the guidelines related to effective debriefing practice.

**Before debriefing:** the establishment by the facilitator of psychological safety and an environment of trust is essential to effective learning using SBL and is a precursor for effective learning [10,11]. Our results imply that an environment of psychological safety is being created in the SBL environments studied and that there is a common understanding between facilitator and students about why debriefing takes place. In an environment where a person feels safe and comfortable, there is a greater openness to development, growth, and negotiating change [12]. Psychological safety is one of the precursors to behavior such as sharing thoughts, asking questions, and asking for help. Creating an environment of trust is essential to students feeling free to express their thoughts, comments, agreements, and disagreements in the interests of meaningful discussions [10]. Facilitators should take meaningful steps to ensure that debriefing environments are considered safe places.

**During debriefing:** learners within a SBL environment aim to perform up to an outcome and to meet an expectation [10]. Linking the debrief to learning outcomes makes it more meaningful to students [13]. This also creates a platform for the student to link theoretical knowledge and cognition. Our results imply that there is a shared mental model and that learning objectives are addressed and that appropriate question techniques are used. This suggests that debriefing is meaningful to students. Meaningful debriefing has the potential to encourage reflection, refine approaches and to channel improvement strategies [14]. Facilitators should be cognisant of the effect that meaningfulness has on student perceptions of debriefing. The way in which a facilitator asks questions has a significant effect on the debrief. Asking open-ended questions encourages engagement, discussion, reflection and self-assessment from simulation participants [10,15-17]. Our results imply that the most common form of questioning is open-ended and not closed-ended. Closed-ended questions are where a simple “Yes” or “No” will suffice and do not generally encourage involvement from students involved in simulation. The questions asked tended to encourage reflection from students and for them to provide meaningful answers about their simulation. The facilitators of debrief should focus on asking questions that encourage engagement, discussion and self-assessment. Debriefing should be conducted in a constructive and encouraging manner [4,6]. Participants in our study did not generally feel embarrassed by the way that debriefing was done. However, almost a third of participants perceived debriefers focusing on what they did wrong rather than what they did right. There is a fine line between debriefing errors in a constructive way and the student perceiving the debrief as focusing on their mistakes. Debriefers should be cognisant of this and remain sensitive to the students when they conduct debriefs. Having a single facilitator for the entire year was the preferred strategy identified by our participants. This may have been due to consistency preferences or may also be due to academic staff constraints. We did not explore this question in greater detail but the implication is that changes in academic staff have the potential to negatively affect.

**Peer debriefing:** peer debriefing is an alternative to instructor-led debriefing. Our results imply that students consider peer-led debriefing a positive practice. One negative aspect related to peer-debriefing is that it has the potential to be unstructured, unlike instructor-led debriefing which tends to be more structured [18]. Peer-led debriefing can be associated with less objectivity of the debriefer and as a result, evaluations may be more generous than those of an instructor may be. Despite the potential risk to objectivity, peer-or self-led debriefing has been shown to be as effective as debriefing conducted by academic staff [19-21]. It should also be considered that the efficacy of peer- or self-led debriefing may be dependent on the ability of the learner to reflect on their experiences [20,21]. Peer- or self-led debriefing is a potentially beneficial debriefing strategy, but should be guided by an instructor to ensure objectivity of the debrief. This study shows that facilitators generally use feedback and debriefing strategies that are linked to good practice. The implication is that the SBL environment is one that is made conducive to learning due to the use of these strategies. There were some areas where improvement was required and these should be considered by facilitators in other domains towards improved practice. The question remains what students in a resource-constrained environment consider as good feedback and debriefing practice. Future research should include a wider sample and the results of this study should be explored from a qualitative African perspective. This may provide important information towards better understanding feedback and debriefing within SBL.

**Limitations:** the study was limited in that despite drawing data from three HEIs, participants were from a single program, limiting the generalizability of the data to the broad healthcare SBL environment. The use of a questionnaire may have resulted in self-reporting bias and it is possible that given the hierarchical nature of the data collection that participant and desirability bias may have affected the results. The use of an English-only paper-based questionnaire may have resulted in cognitive bias based on how the participants understood the questions.

## Conclusion

Several strategies related to effective feedback and debriefing were identified by the student participants as already being employed by facilitators. The potentially negative effect of multiple facilitators was highlighted by participants who indicated that they preferred a single debriefer for the entire academic year. Peer-led debriefing was perceived as a positive practice and has a number of advantages and disadvantages that should be considered and mitigated by the facilitator. Negative practices identified by participants included focusing on what a student did wrong as opposed to what they did right and asking binary “Yes/No” type questions.

### What is known about this topic

- Debriefing is a critical component of healthcare simulation;
- Effective debrief strategies have been shown to enhance performance and ineffective strategies can negatively affect student performance;
- There are certain practices within debriefing that directly affect how effective the debriefing is.

### What this study adds

- Current feedback and debriefing practices at the HEIs surveyed generally followed principles of positive feedback and debriefing;
- Participants viewed peer-debriefing as a positive strategy and preferred the consistency of a single facilitator for the academic year- these should be considered in the SBL classroom;
- Focusing on what the student did wrong as opposed to what they did right was more common than anticipated and SBL classroom practice should actively aim to limit this.
